# *In vivo* Analysis of Choroid Plexus Morphogenesis in Zebrafish

**DOI:** 10.1371/journal.pone.0003090

**Published:** 2008-09-01

**Authors:** Marta García-Lecea, Igor Kondrychyn, Steven H. Fong, Zhang-Rui Ye, Vladimir Korzh

**Affiliations:** Cancer and Developmental Cell Biology Division, Institute of Molecular and Cell Biology, A-STAR, Singapore, Singapore; Centre de Regulacio Genomica, Spain

## Abstract

**Background:**

The choroid plexus (ChP), a component of the blood-brain barrier (BBB), produces the cerebrospinal fluid (CSF) and as a result plays a role in (i) protecting and nurturing the brain as well as (ii) in coordinating neuronal migration during neurodevelopment. Until now ChP development was not analyzed in living vertebrates due to technical problems.

**Methodology/Principal Findings:**

We have analyzed the formation of the fourth ventricle ChP of zebrafish in the GFP-tagged enhancer trap transgenic line SqET33-E20 (Gateways) by a combination of *in vivo* imaging, histology and mutant analysis. This process includes the formation of the *tela choroidea* (TC), the recruitment of cells from rhombic lips and, finally, the coalescence of TC resulting in formation of ChP. In Notch-deficient *mib* mutants the first phase of this process is affected with premature GFP expression, deficient cell recruitment into TC and abnormal patterning of ChP. In Hedgehog-deficient *smu* mutants the second phase of the ChP morphogenesis lacks cell recruitment and TC cells undergo apoptosis.

**Conclusions/Significance:**

This study is the first to demonstrate the formation of ChP *in vivo* revealing a role of Notch and Hedgehog signalling pathways during different developmental phases of this process.

## Introduction

The choroid plexus (ChP) represents a thin outgrowth of the dorsal midline ependyma into the brain ventricles. Its main function is to produce the cerebrospinal fluid (CSF) that plays an integral role in normal brain function and development as well as in recovery from injury [1], [2]. The morphogenesis of ChP has been studied in amniotes mainly in the lateral ventricles [3]–[5] and some studies reported development of the fourth ventricle ChP [6]–[8]. As a result four stages of ChP development have been defined on the basis of the morphology of epithelial cells and the presence of glycogen, whose function is unknown [3], [9]. The fourth ventricle ChP arises from precursors that also give rise to roof plate and astrocytes [10]–[12]. In contrast, the developmental analysis of ChP in anamniotes has been limited to amphibians [13], and the comparative anatomical studies of adults represent the bulk of information about ChP in fish and amphibians [14]. In the absence of lateral ventricles, teleosts feature ChP in the third and fourth ventricles only [15].

Hedgehog (Hh) and Notch signalling pathways participate in many developmental events in vertebrates. Notch signaling plays a role in cell fate decisions in many different tissues of multicellular organisms including the nervous system. In most cases, Notch blocks primary differentiation fate and directs cells to a second, alternative differentiation program or keeps them undifferentiated. In contrast Delta, a ligand for Notch receptor, forces cells towards primary differentiation fate [16], [17]. The Hh signalling promotes survival and proliferation of neural progenitors in the ventral neural tube and prevents their apoptosis. These events take place prior to the regional specification of cells, which happens independently of the D-V specification that Hh is largely known for [18], [19].

Some components of these pathways have been implicated in normal development and pathology of the ChP [20]–[24]. Several zebrafish mutants disrupt these signalling pathways: for example, *smu* blocks the Hh signalling [25]–[27] and *mib* - the Notch signalling [28]–[31].

The transposon-mediated enhancer-trap (ET) identifies genomic regions regulating developmental genes [32]. The transposase-deficient *Tol2* transposon vector carrying the enhanced green fluorescent protein (*EGFP)* gene under the control of a partial promoter of the simple epithelia-specific *keratin4* (ZFIN – zebrafish information network) was used for such screen generating a number of transgenics [33], [34]. One of these - SqET33 - was further used as “a launching pad” for transposon jumps into new sites after injection of transposase mRNA, which resulted in the generation of SqET33-E20 (renamed as Gateways). The Gateways transgenics demonstrated a characteristic and strong GFP expression in several sites along the dorsal midline of the neural tube including the roof of hindbrain, where ChP develops. We used these transgenics to describe *in vivo* development of the fourth ventricle ChP of zebrafish during normal development and in mutant embryos affecting Hh and Notch signalling pathways and revealed requirements for these pathways during different phases of ChP development.

A similar approach has been taken in a parallel study using another transgenic line, Et^Mn16^ [35], which starts to express GFP in the ChP slightly later compared to Gateways.

## Results

### Characterization of the transgenic line Gateways

**Figure 1:**
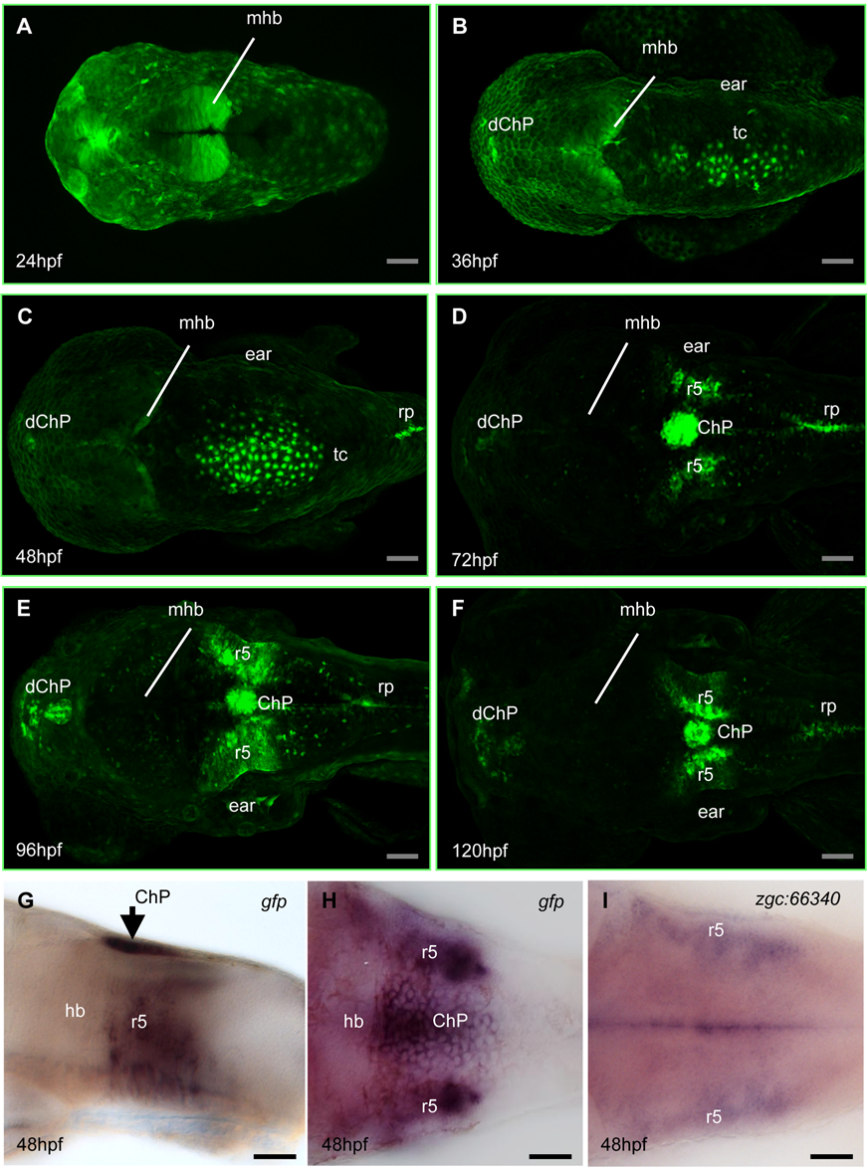
Formation of the fourth ventricle choroid plexus in Gateways zebrafish transgenics. A, at 24hpf GFP expression was absent at the roof of fourth ventricle; B, at 36hpf GFP-positive cells are present at the dorsal midline of the roof of fourth ventricle; C, the number of GFP-positive cells increased, they are separated from the roof plate posteriorly; D, ChP has formed at the dorsal midline at the ear level; E, F, ChP tightens. G, H, lateral and dorsal view of *gfp* expression pattern in transgenics. Compare that to i showing expression of *zgc:66340* in dorsal view. A–F, dorsal view. Abbreviations: dChP – diencephalic ChP; ChP – choroid plexus of hindbrain, hb – hindbrain, mhb – midbrain-hindbrain boundary, r5 – rhombomere 5, rp – roof plate, tc – tela choroidea. In all figures scale bars = 50 µm unless otherwise indicated.

SqET33-E20 (Gateways) is one of the enhancer-trap (ET) lines generated by the remobilization of Tol2 transposon in a primary ET transgenics - SqET33 (chr. 14) [33]. The Gateways homozygotes are fertile. They carry a single insertion in Chr. 24 within a region containing several genes whose expression pattern was unknown or, if available, not informative. In the CNS of Gateways GFP is expressed in the dorsal and ventral diencephalon, rhombomere 5 (r5), neurons along the midline of ventral hindbrain, dorsal midline of hindbrain, roof plate, lenses, olfactory pits, branchial arches and ears (Fig. 1 and not shown). To characterize the regulation of *gfp* expression in detail, we cloned several genes located close to the insertion: *zgc:66340* similar to *axud1* (*Axin1 up-regulated gene 1* or *Csrnp1*) [36]–[37], wu:fk14e08 – *sulfatase FP1c* , zgc:153639 – similar to solute carrier organic anion transporter family member 22A14 (SLC22A14), *ENSDARG00000071685* – SLC5A1, ENSDARESTG00000006431- similar to cardiomyopathy associated protein 1, zgc:55494 - serine/threonine-protein kinase OXSR1b (Fig. S1A). Unfortunately, their expression was either too low or recapitulated *gfp* expression only partially and none was expressed in the ChP at detectable levels (for example, Fig. 1I). Thus a more detailed analysis within a larger genomic region is probably needed to find a gene with expression pattern that recapitulates the one of *gfp* in the ChP. In parallel, we identified several other transgenic lines with insertions in the same genomic region of Chr. 24 that were used as independent markers (Fig. S1B). In two of these lines GFP was expressed in the ChP indicating that the genomic region where they reside is controlled by the same regulatory element that regulates gene expression in the ChP of both diencephalon and hindbrain (Fig. S1C-F).

### GFP-positive cells in the roof of fourth ventricle represent the choroid plexus

Interestingly, the midline domain in the roof of the fourth ventricle of Gateways embryos and larvae is in position of ChP [15], [38]. This opened the possibility to study the morphogenesis of ChP *in vivo*. At 24hpf GFP expression was present at the midbrain-hindbrain boundary (mhb), epiphysis and in olfactory placodes (Fig. 1A). At 29hpf a group of GFP-positive cells appeared at the roof of the fourth ventricle (not shown) and as the GFP expression and number of cells increased, at 36hpf these cells formed a sheet close to the midline (Fig. 1B), which probably represents the ChP primordium - *tela choroidea* (TC). At 48hpf, the TC is separated from roof plate posteriorly and from the rhombic lips laterally and anteriorly (Fig. 1C). Between 72hpf and 144hpf the TC coalesced into a single domain in the position of the fourth ventricle ChP (Fig. 1D–F). This GFP expression is recapitulated at the RNA level (Fig. 1G,H).

**Figure 2:**
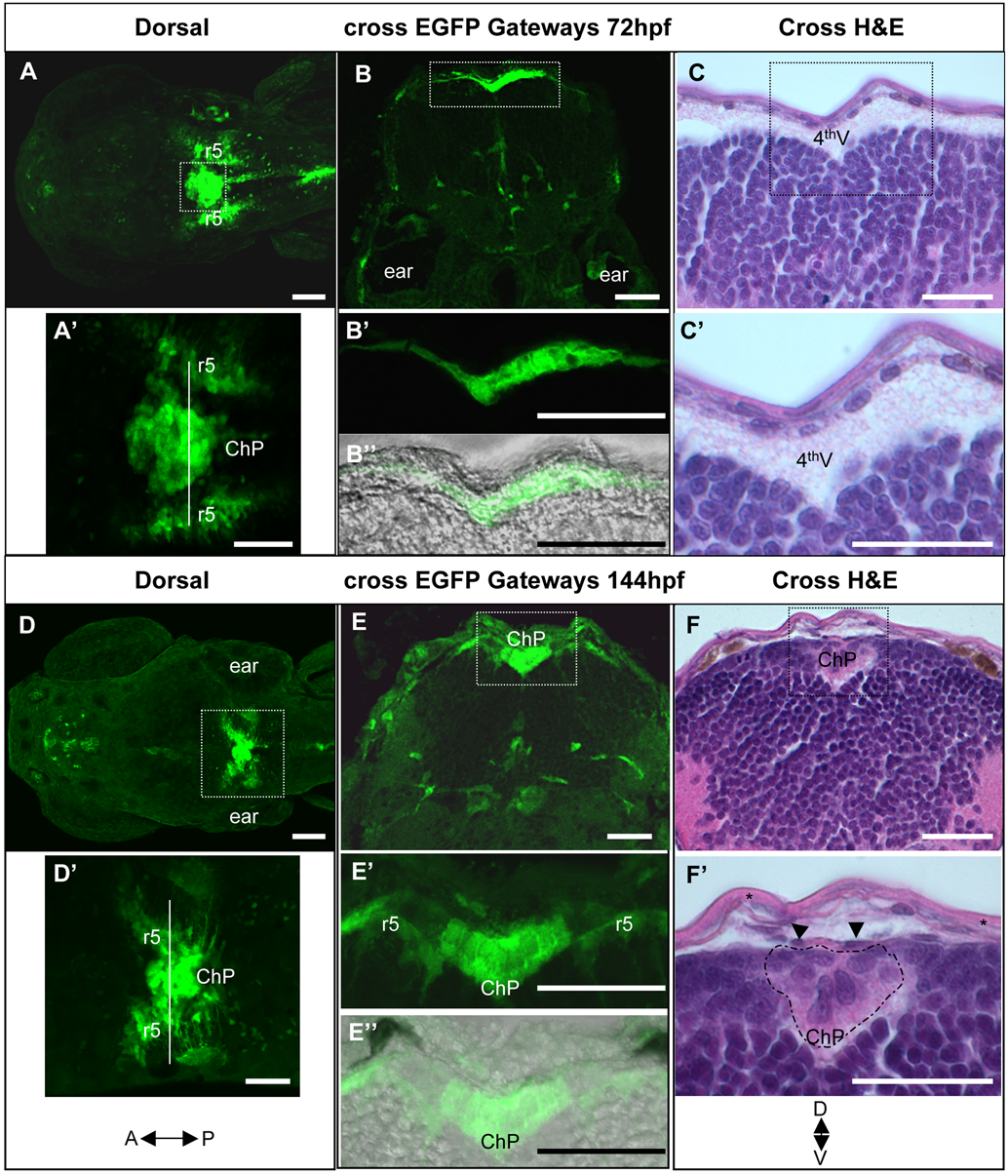
Comparative histological and developmental analysis of the fourth ventricle choroid plexus in Gateways zebrafish transgenics. A–C – 72hpf, D–F – 144hpf. A, A', D, D', - dorsal view, B, B', B”, C, C', E, E', E”, F, F' – cross sections. All A, B, D, E images show GFP expression, all C and F bright field images show staining with hematoxylin-eosin; B” and E” are composite fluorescence-bright-field images. Abbreviations: ChP – choroid plexus, 4^th^V – fourth ventricle, r5 – rhombomere 5, starlet in F' – skin epithelium, arrowheads in F' – vessel.

At 72hpf two GFP positive domains were found at both sides of the developing ChP at the level of the ears (Figs. 1D, 2A,A'). The GFP-positive radial cells present in both clusters organized laterally in rhombic lips at the level of r5 and remained in that position keeping close contact with the ChP (Fig. 1D–H, 2A', D', E' and movie S1).

We analysed cross sections stained by anti-GFP antibody and haematoxylin-eosin (HE). Prior to formation of the dorsal midline cluster the GFP-positive oval-shaped cells formed a monolayer immediately beneath the epithelium (Fig. 2A–C). Ventrally they faced the fourth ventricle containing some lightly coloured substance (Fig. 2C). At 144hpf the cluster formed the characteristic raceme-like structure with large oval-shaped cells that are clearly distinct from the ventral small and round neural cells and the dorsal flat elongated endothelial cells (ECs, Fig. 2E-E”, F, F'). By this stage the ventricle had diminished and the cluster occupied most of it being closely associated on both sides with the bilateral clusters of GFP-positive radial cells in r5 (Fig. 2E'). Interestingly, blood vessels were not detected within the cluster similar to that in other teleosts [39].

The anatomical position and results of our histological analysis suggested that the GFP-positive cluster in the roof of fourth ventricle indeed represents ChP.

### Lineage of choroid plexus

**Figure 3:**
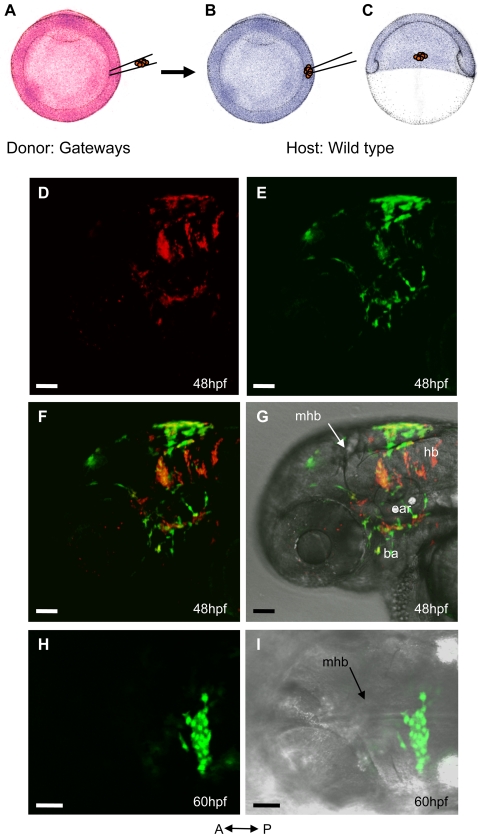
Lineage analysis of ChP. Transplantation was performed at 6hpf and the outcome was visualized at 48hpf (n = 5) and 60hpf (n = 4). A–C, schematic of the transplantation experiment. A,B, drawing of the animal view of 6hpf embryos. C, drawing of the lateral view of a 6hpf embryo. A, Texas Red-injected Gateways donor. B,C, wild type host. D–G, lateral view of 48hpf host embryo. D, Texas Red-labelled descendants of transplanted cells. E, GFP positive descendants of transplanted cells. F, merged D&E. G, merged F & bright field image. H,I, dorsal view of 60hpf host embryo. H, cluster of GFP-positive cells in the roof of fourth ventricle. I, merged H & bright field image. Abbreviations: ba – branchial arch, hb – hindbrain, mhb - midbrain-hindbrain boundary.

Cells in the ChP and bilateral group of r5 are close by and express GFP suggesting that these cell types could be related. To verify this idea, we performed transplantation of Texas Red-labelled transgenic cells from the lateral neural plate of Gateways into the same position of unlabelled control embryos at 6hpf (Fig. 3A–C). At 48hpf this resulted in appearance of Texas Red/GFP-positive cells at the roof of dorsal midline and r5 of host embryos (Fig. 3D–G). Later on these cells formed a tight cluster at the dorsal midline reminiscent of the ChP (Fig. 3H, I). Some scattered GFP-positive cells were also found in branchial arches and ear capsule suggesting that they derived from neural crest (Fig. 3G and compare to Fig. S1F).

### Choroid plexus and vasculogenesis

**Figure 4:**
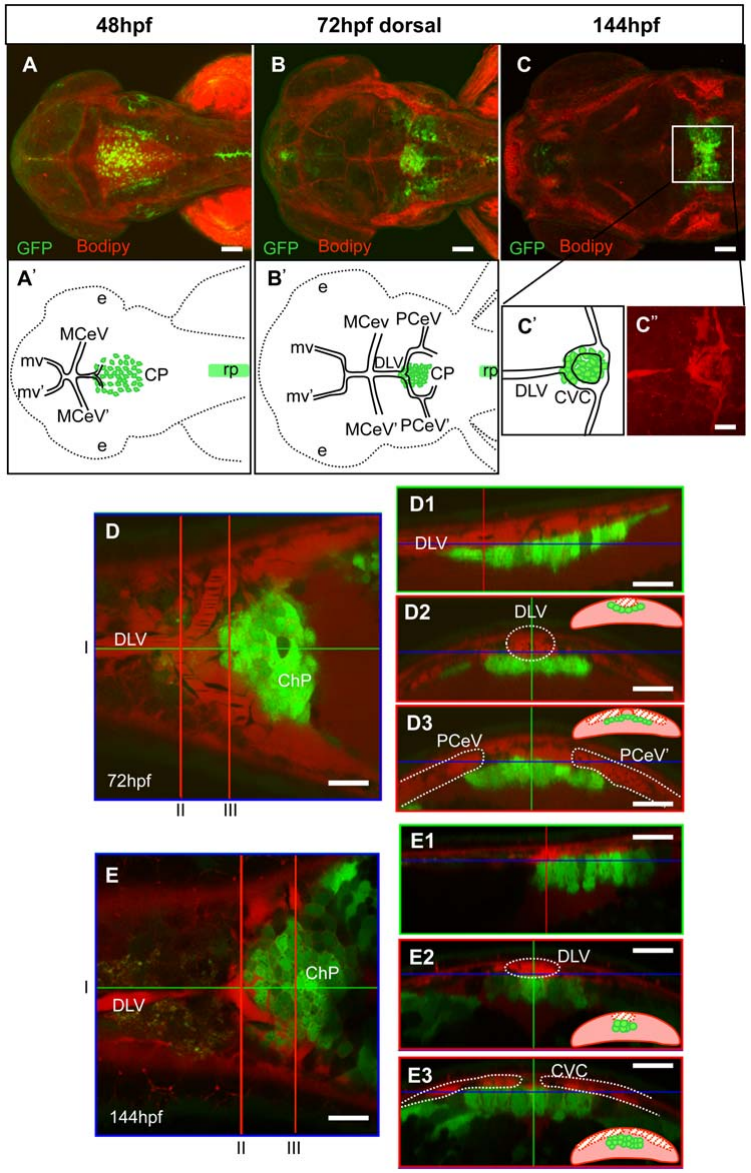
Comparative analysis of formation of the fourth ventricle ChP and vasculogenesis. Staining of Gateways by Bodipy (red) allows the *in vivo* analysis of ChP and cranial vasculature development. Extension of the DLV, growth of PCeV and closure of CCV occur at the same time as the transformation of the *tela choroidea* into ChP. A–C, confocal images at three different time points with their respective explicative drawings (A', B', C', C”). D, E - dorsal optical section focused on ChP; D1 and E1 saggital optical sections at the level of the green line in D, E; D2, D3, E2, E3 cross optical sections at the level of the red lines in D, E. At all times ChP keeps a close contact with the developing vessels. Abbreviations: ChP – choroid plexus, CVC – choroidal vascular circuit, dlv- dorsal longitudinal vein, mv- midbrain vein, PCeV- posterior cerebral vein, rp- roof plate. Scale bar in D, D1–D3, E, E1–E3, 20 µm.

ChP performs many functions, including production of CSF [1]–[3]. This requires an interaction with brain vasculature. To find when that interaction takes place we have used Cell Trace™ Bodipy® TR methyl ester as a live marker of the cranial vessels. It seems that the developing ChP makes close contact with ECs and hindbrain cranial vessels early (Figs. 2F' & 4). At 48hpf both the ChP and dorsal cranial vessels slightly overlap in dorsal projection. At this time, (i) the middle cerebral veins (MCeV) have developed in the dorsal midbrain, and (ii) the dorsal longitudinal vein (DLV) sprouts towards the hindbrain after the appearance of the GFP-positive population of cells but prior to completion of ChP morphogenesis [40] (Fig. 4A, A'). Later on the posterior cerebral veins (PCeV) branch from the DLV and ChP cells coalesce beneath that branching point in close contact with the vessels (Fig. 4B, B', D). At 144hpf PCeVs surround the ChP and branch away from its right and left sides (Fig 4C, C', C”, E). As the ChP is confined within that vascular circuit we have named it Choroidal Vascular Circuit (CVC; Fig 4C', C”) to differentiate it from the previously named choroidal vascular plexuses (CVP) [40] that form bilaterally in the midbrain.

### *In vivo* imaging of ChP formation

To understand the formation of ChP in detail, we made *in vivo* time-lapse movies that defined three phases of ChP development.

During the first phase (29hpf–36hpf) GFP-positive cells appeared in the roof of the fourth ventricle forming the TC (Fig. 1B). Some of the GFP-positive cells were already at the dorsal midline when detected (yellow traced cells, movie S2), whereas others emerged from rhombic lips (pink traced cells, movie S2).

During the second phase (36hpf–48hpf) recruitment of cells from rhombic lips into TC continued (Fig. 1B–D; see also green arrows and pink traced cells in movie S3). Importantly, none of GFP-positive cells got fragmented. The TC and spinal roof plate were separated. The TC was displaced anteriorly along with the whole neural tube and ears. Cells moved a little with respect to each other within the sheet. This differed from the active movement of neighbouring cells that “patrolled” the edge of TC (red dot traced cells in movie S3). The DLV took form through this second phase and at this point started branching into the PCeV demarcating the prospective ChP area (see Fig. 4A).

**Figure 5:**
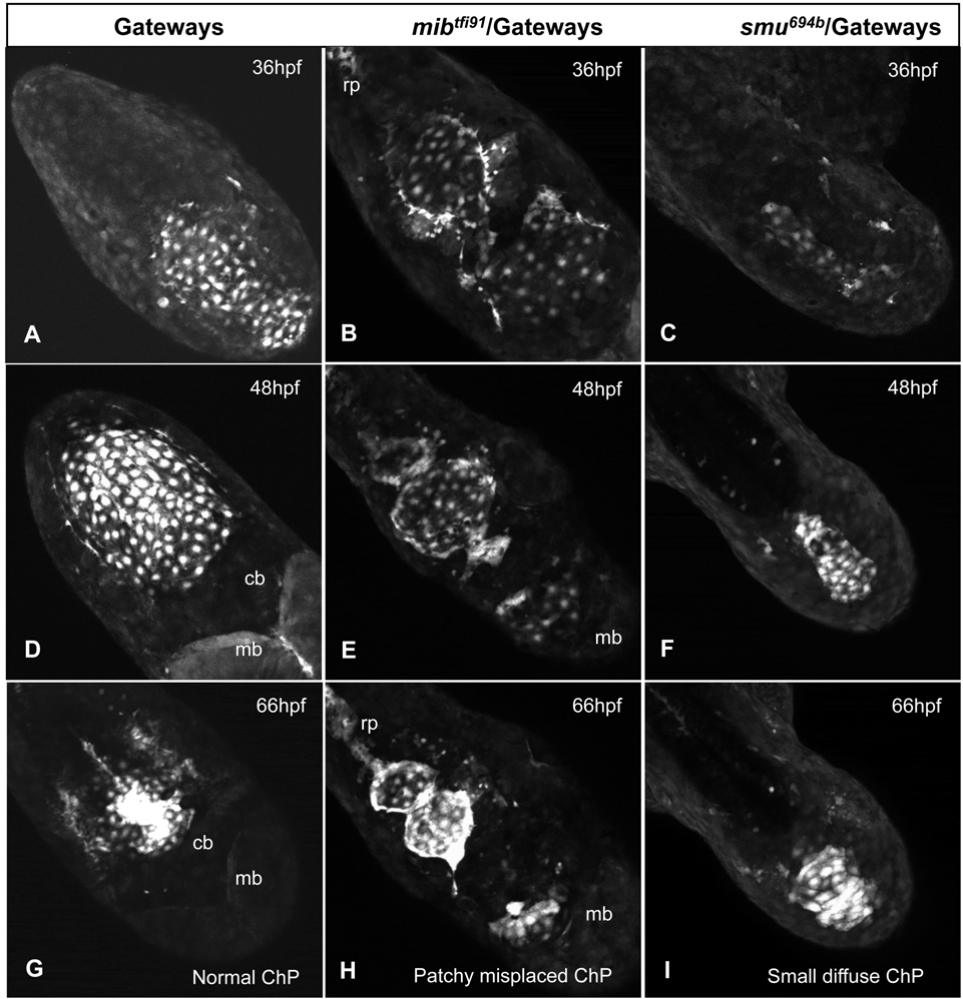
Mutant analysis of the formation of the fourth ventricle ChP. A, D, G – control; B, E, H, - *mib^tfi91^*; C, F, I - *smu^694b^*. A–C – 36hpf, D–F – 48hpf, G-I – 66hpf. Images are confocal z-projections extracted from the movies. All images are taken in dorsal view with the anterior part of the embryo towards the right-bottom corner. In controls and *mib* mutants dividing cells were detected and none of cells were fragmented. In *smu* a number of fragmenting cells were detected (purple arrow, movies) with no cells dividing. Abbreviations: cb – cerebellum, mb – midbrain, rp- roof plate.

During the third phase (48hpf–144hpf) GFP-positive cells initiated active movements towards the middle position at the ear level and TC slowly transformed into distinct raceme-like ChP (Fig. 1D–F, 5G and see movie S4; 66–144hpf not shown). By 144hpf (6dpf) ChP was ventral to the CVC and in close contact with this vessel (Fig. 4C,E).

Taken together, the *in vivo* observations (movies S1, S2, S3 and S4) and cell transplantation (Fig. 3) demonstrated that at least some cells of the lateral neural plate give rise to the precursors in rhombic lips, which in turn give rise to cells in the ChP and r5.

### Notch signalling and ChP formation

In the *mind bomb* (*mib*) mutant the E3 ubiquitin ligase is defective causing premature differentiation of neural progenitors [29]–[31], [41]. The development of ChP in Gateways/*mib^tfi91^* is summarized in Figure 5 and displayed in movies S4 and S5.

**Figure 6:**
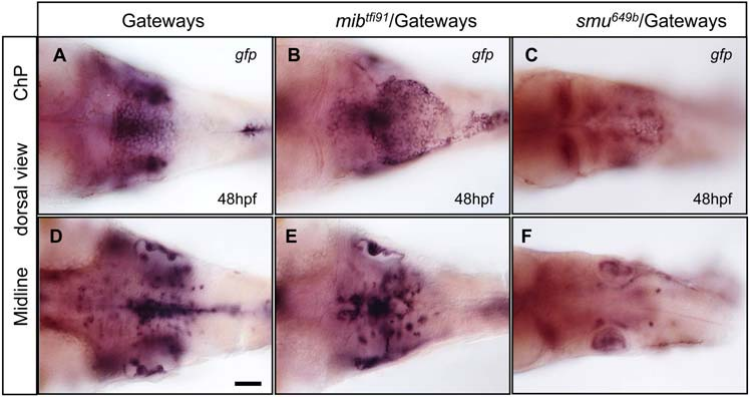
ChP and GFP-positive neurons at the ventral midline are affected in mutant transgenics as detected by anti-*gfp* WISH. A, D – control, B, E - *mib^tfi91^*, C, F - *smu^694b^*. A–C – ChP, D–F – ventral midline. All images show *gfp* mRNA expression and are in dorsal view, anterior to the left.

In *mib^tfi91^*/Gateways embryos, ChP is affected. GFP-positive cells appeared at the dorsal midline prior to 28hpf. From 36hpf to 54hpf several cell division events were detected in the mutant (orange cells traced in movie S5). Overall, in this *mib^tfi91^*/Gateways embryo (movie S5) four cells divided within the TC. Similar to controls, no cells became fragmented. The number of GFP-positive cells in the mutant TC did not significantly differ from normal (Table 1). The TC in *mib* was abnormally patterned with a “black belt” of GFP-negative cells forming across the ventricle (Fig. 5B, E, H). Many GFP-positive cells were attached to rhombic lips unlike that in controls. The anterior and posterior limits of the TC were not respected with some cells invading the territory adjacent to cerebellum and others connecting to roof plate posteriorly (Fig. 5E, H; Fig. 6B and see blue trace in movies S5 and S6). Eventually TC cells formed several small aggregates along the dorsal midline of the ventricle (Fig. 5H and movie S6). All this is in line with the global patterning defects in the hindbrain of *mib* suggested earlier [28], [30], [41] and indicates that proper formation and position of ChP depend on Notch signalling as demonstrated by functional analysis of Notch signalling [35].

**Table 1:** Comparative analysis of GFP positive cells in TC at 36hpf.

Embryo	GFP-positive cells		
	*wt*	*mib*	*smu*
1	52	57	16
2	56	56	35
3	66	58	28
4	65	51	20
Average (n = 4)	60±7	56±3	25±8

### Hh signalling regulates cell number in ChP

In the *smu^b641^* mutant the Hh signaling is blocked resulting in the absence of several populations of ventral neurons, lateral floor plate, parts of ventral forebrain, pituitary placode and defects in the optic chiasm and ventral forebrain commissural axon tracts [25], [26].

We have analysed the formation of ChP in *smu^b641^*/Gateways. GFP-positive cells appeared with delay (30–31hpf), their number was reduced and they formed a narrow stripe at the dorsal midline (Fig. 5C). The recruitment of cells was affected as if the second phase of ChP morphogenesis was missing (Fig. 5C, F, I). At 48hpf *gfp* expression at mRNA level was substantially reduced (Fig. 6C,F). In *smu^b641^*/Gateways cells were larger and far less in number (Table 1), whereas the ChP when formed was smaller and less compact than in controls. We did not notice any cell divisions in the movies. This could be due to an increased embryo curvature, which made observations less precise. Indeed, the overall mitotic activity in the mutants did not differ from normal embryos as shown by anti-pH3 staining (Fig. S3). On the other hand five cases of cell fragmentation were detected during the second phase of ChP formation (movie S7) and at least one during the last phase (movie S8) illustrating cell death in the mutant TC. Moreover TUNEL staining supports these observations as it shows an increased apoptosis of CP cells in *smu* mutants (Fig. S2 and Table 1).

In conclusion, we used the Gateways transgenics as a living marker to describe for the first time the *in vivo* morphogenesis of ChP of the fourth ventricle and defined three characteristic phases of this process. Using lineage tracing, we demonstrated that the ChP originates from the lateral neural plate and shares its origin with radial cells of r5 and some neural crest derivatives. We also analysed the formation of ChP in mutants affecting the Notch and Hh signalling pathways and defined specific developmental defects of ChP caused by genetic abnormalities affecting these pathways.

## Discussion

Many studies have described various aspects of ChP physiology [1] and its development in amniotes using traditional histological analysis of fixed material [3]. Lately, these approaches were expanded to include transgenic mammals [10], [11], [20]. Recently, the *in vitro* culture of ChP after dissociation of isolated porcine ChP tissue has been developed to study the protein-mediated transport of drugs from blood to CSF. This model probably better reflects a situation of reassembly of differentiated cells similar to ChP regeneration following injury of adult brain [42], whereas Gateways embryos could be more useful for similar studies focused on normal development.

Whereas the ChP in teleosts has been identified, no developmental studies have been made [15]. The *in vivo* analysis of ChP formation in vertebrates is not available either. Our study fills both these gaps and for the first time provides a detailed *in vivo* description of ChP formation in vertebrates. Similar to that described in other species the GFP-positive ChP of zebrafish as expected develops at the dorsal midline of the fourth ventricle in close contact with dorsal cranial vessels. We did not observe sprouting of capillaries from the dorsal veins into the ChP proper as late as 144hpf, which implies it either takes place during later development or otherwise indicates a simpler organization of the ChP-associated vasculature in zebrafish compared to that in mammals, where fenestrated capillaries are embedded in the ChP proper. The second suggestion is in line with studies in other teleosts [39].

Using to our advantage the transparency of zebrafish embryos and early larvae we performed a detailed *in vivo* analysis of ChP formation and as a result defined three phases of ChP morphogenesis based on cell migration events and mutant analysis. These phases are different from those defined earlier for sheep and human embryos, which were based mainly on the morphology of epithelial cells and the presence of glycogen [3], [9].

### The nascent TC is disrupted in *mib* mutants

The first phase (29–36hpf) represents the appearance of the TC. It is characterized by both the recruitment of cells originated from the dorsal neuroepithelium and migration of cells from rhombic lips towards the midline where they form the monolayered sheet of TC as manifested by an appearance of GFP-positive cells at the dorsal midline. This phase is abnormal in *mib* mutants, where cell differentiation starts prematurely, the ability of cells to join the TC is impaired and the sheet of GFP-positive TC is interrupted by GFP-negative cells. This early disruption of TC rises a number of questions such as what regulates the initial assembly of TC, and whether this property is intrinsic or extrinsic to the TC. The abnormality of the ChP in *mib* is caused by an early defect in its patterning. This could be related to the inherent heterogeneity of the TC and presence of several distinct populations of cells [10].

It has been shown that migration and assembly of various primordia depend on expression of proteins that mediate differential cell adhesion events, and also that differentiated cell lines of ChP vary in expression of these proteins [43]–[45]. The increased level of Delta in *mib* mutants has been seen to potentially stimulate a clustering behaviour of cells [29], [46], which is in line with abnormal accumulation of GFP-positive cells at the *mib* rhombic lip. Thus, it is possible that maintenance of proper levels of Notch signalling regulates coalescence of ChP. At the same time it has been shown in mammals that the ChP is assembled from more than one cell type [10]. Taking into account that Notch-Delta interactions are known to generate heterogeneity of cells it is possible that similar events take place during formation of the TC [29], [30], [41]. If so, one of the cell types could act as an intrinsic regulator of ChP coalescence. Given that ChP cells in Gateways acquire GFP expression in the midline population earlier than cells in Et^Mn16^ [35], it is tempting to speculate that these transgenic lines reveal two different populations of ChP cells. More detailed analysis of these transgenics will be required to support this idea. Importantly, at the end of ChP morphogenesis the midline group of cells is connected to the bilateral groups of radial cells in rhombic lips of r5 (Fig. 2E'). It is not clear whether this event represents one more stage of ChP morphogenesis and whether the bilateral clusters represent a separate population of ChP cells. It has been suggested that in mammals ChP starts to form proximal to rhombic lips [11]. Perhaps, this scenario better reflects the developmental events that take place in Et^Mn16^, where GFP expression appears laterally in cells of r5 earlier than in the midline ChP [35]. If this were the case, our data showing, in Gateways, an early expression of GFP in the midline population of cells and its later assembly adds a new dimension to our views of the formation of ChP in vertebrates. Clearly more work is needed to see whether this idea is correct.

### Hedgehog signalling is required to maintain viability of TC cells

The second phase is characterized by the recruitment of cells from rhombic lips into the TC. This process is disrupted in *smu* mutants that lack Hh signalling acting to promote cell proliferation and to repress apoptosis in the ventral neural tube [18], [19], [26]. And yet it was shown that this signalling also plays an antiapoptotic role in dorsal progenitors [26], [47]. In this context our recent demonstration of a role of Gli2 proteins in proliferation of progenitors in the dorsal hindbrain of zebrafish supports this idea [27]. Furthermore, ECM proteins play a key role in presenting Hh to their target cells and can also actively regulate Hh signalling in CNS [48], [49]. Interestingly, our histological analysis shows the light pink ECM within the fourth ventricle (Fig. 2C', F'). Indeed the CSF contains some ECM molecules (vitronectin and fibronectin) [50]. So it is plausible that Hh produced at the ventral midline could reach the ChP region through diffusion or its interaction with ECM proteins (or both). Thus the reduced number of cells in the ChP of *smu* could be due to an apoptosis that peaks during early stages of ChP formation in absence of Hh (movies S7–S8, Table 1 and Fig. S2). These results open the opportunity to study events of Hh-dependent apoptosis *in vivo* focusing on specialized cell lineages or organs, in particular in the ChP. Since Hh deficiency causes apoptosis in many cell types, including neural crest [47] and *locus coeruleus* [51], an availability of transgenics with expression of fluorescent reporters in particular cell lineages and organs may help to develop a better understanding of specific needs of different cell lineages in this morphogen,

### ChP forms due to cell coalescence

The third phase (48–144hpf) represents assembly of the ChP due to an active coalescence of cells. Such activation of cell movement suggests a major change in cell behaviour manifesting a different developmental mechanism involving probably the acquisition of the ability to sense the place of coalescence and being able to move in a coordinated manner not only along the medio-lateral axis, but also towards a specific position along the anterior-posterior axis. Perhaps the fast movement detected is linked to the appearance of the glycogen stores. Future experiments could evaluate whether glycogen in zebrafish ChP is distributed in a similar fashion to that in other vertebrates [3], [9]. The presence of glycogen may provide a mechanistic explanation of how cells develop the ability to move fast during the final phase of ChP morphogenesis.

### Cell lineage of ChP cells

It was previously described that different cell types contribute to the mammalian ChP. Some of those cells share their origin with hindbrain roof plate and others originate directly from the rhombic lips [10]. The question remains whether the same developmental scenario takes place in zebrafish. Our results show that indeed cells delaminating from the rhombic lips contribute to the ChP field, but the analysis of movies was not informative to trace origin of ChP cells. The transplantation experiments demonstrated a common origin of the ChP, cells in bilateral clusters of r5 and neural crest derivatives. Therefore, we can conclude that in zebrafish ChP cells derive from the lateral edge of the neural plate. But given the fact that more work is needed to define different cell lineages within the ChP, it could be premature to conclude that all cell types in the ChP derive from the lateral neural plate.

## Materials and Methods

### Animals

Zebrafish were maintained according to established protocols [52] in agreement with the IACUC regulations and rules of the IMCB zebrafish facility. Embryos were staged in hours (hpf) or days (dpf) post fertilization. The mutant alleles are *mib* ^tfi91^ [28], *smu* ^b641^ [25].

### Live imaging and movies

The pigmentation was inhibited with 0.2 mM 1-phenyl-2-thiourea (PTU) in egg water. For *in vivo* imaging embryos were dechorionated at the selected stages, anaesthetized with 0.2% tricaine and oriented by embedding them in 0.8% low melting agarose (LMA) in embryo water on a glass coverslip floor of a small petri dish plate. While the agarose was still liquid, embryos were positioned with two needles and left for 5–10 min at room temperature until agarose set and was hard enough to hold the embryo. ChP was best seen in dorsal projection. For time-lapse recording the embedded embryos were covered with embryo water containing PTU and tricaine and maintained in a thermostated chamber at 28°C for 16–18 h periods . All embryos held in the imaging chamber maintained heartbeat and circulation throughout the imaging period. Separate frames each of which represents the 3D reconstruction of confocal z-stacks recorded with 15 min intervals were assembled into final movie.

Microscopic observations were done using a dissecting fluorescent microscope SZX12 (Olympus, Japan) and a compound microscope Zeiss Axioscope2. Still images were captured with an inverted Zeiss LSM 510 (Zeiss, Germany) and time-lapse movies of live zebrafish embryos with Olympus Fluoview (Olympus, Japan). 2D or 3D reconstructions of image data were prepared using the standard LSM or Olympus software package. Digital images of live embryos were processed with Photoshop (Adobe Systems, USA) and time-lapse series were processed with ImageJ (NIH), adjusting background brightness and contrast.

CellTraceTM Bodipy TR methyl ester is a vital fluorescent dye that permeates cell membranes and is ideal to detect shapes of cells as well as boundaries of organs. It also accumulates in extracellular space, which makes it a useful tool to reveal *in vivo* blood vessels and body cavities like the brain ventricles. Prior to agarose mounting and imaging, the embryos were incubated for 30 min in 100 µM Bodipy prepared in embryo water at room temperature and then washed three times with embryo water.

### Whole mount in situ hybridization (WISH), fluorescent immunohistochemistry and histology

Embryos were processed for WISH, cryosectioning and immunohistochemistry as before [53]. The following antibodies were used: mouse monoclonal anti-GFP antibodies (clone B-2, Santa Cruz Biotechnology, 1 µg/ml) and goat anti-mouse/Alexa Fluo488 (Molecular Probes, 2 µg/ml). For haemotoxylin-eosin staining, embryos fixed with Bouin's fixative were mounted in bactoagar blocks, processed into wax and sectioned following standard histological protocols.

### Whole mount immunochemistry and TUNEL

For TUNEL staining embryos were processed as described in [54]. Apoptosis was detected with the In situ cell death Detection Kit, TRM red (Roche). GFP/pH3 double labelling was performed using the following primary antibodies: polyclonal anti-phospho Histone H3 (1 µg/ml; Upstate, Lake Placid, NY) and mouse monoclonal anti-GFP antibodies (clone B-2, Santa Cruz Biotechnology, 1 µg/ml). Secondary antibodies were goat anti-mouse/Alexa Fluo488 and goat anti-rabbit/AlexaFluo633 (Molecular Probes, 2 µg/ml).

### Enhancer trap lines Tol2

The generation of the first population of enhancer trap transgenic zebrafish, their phenotypes and the remobilization of the Tol2 transposon from SqET33 has been described elsewhere [33].

### Cell transplantation

Cell transplantation was performed as described in [43]. In brief, 1–2 cell stage Gateways transgenic embryos were injected with Texas Red-dextran. At 6hpf 5–10 cells of the lateral neural plate were transplanted into the same position in unlabelled control embryos of the same stage, which developed normally. At 48hpf and 60hpf embryos were fixed and processed for two-colour detection of GFP and Texas Red.

## Supporting Information

Figure S1 Characterization of the Gateways Tol2 insertion site. (A) 600-kb locus of Chr. 24 (Zv7, release 49). Triangles represent ET insertions, where green triangles show insertions with GFP expression in ChP. (B) zgc:66340 locus. TSS at 12251781 bp was determined using 5′-RACE. The coding exons are depicted as black boxes, untranslated regions are represented as open boxes. The ET insertions are shown according to their position in zgc:66340 locus, green arrow shows EGFP gene in the ET construct (direction of the reporter gene transcription corresponds to the direction of arrow), gray box is a minimal promoter, dotted arrows represent 5′- and 3′-ends of Tol2 transposon). ET, enhancer trap; TSS, transcription start site; EGFP, enhanced GFP. (C,D, C',D') Dorsal and lateral views of GW42A and Gateways lines with insertions located in promoter region at different distances from TSS exhibit GFP expression in ChP. GW45C line with insertion located in intron has a weak GFP expression in ChP (not shown), while another intron-based insertion GW42B shows background GFP expression in skin (not shown). Abbreviations: ba - branchial arch, dChP - diencephalic ChP; ChP - choroid plexus of hindbrain, r5 - rhombomere 5, rp - roof plate.
(2.50 MB TIF)

Figure S2 Apoptosis increased in smu mutant as detected by TUNEL. Two developmental stages were analyzed: 36hpf (A–C Gateways, D–F smu694b/Gateways) and 48hfp (G–I Gateways, J–L smu694b/Gateways). A,D,G,J - TUNEL; B,E,H,K - anti-GFP antibody staining; C,F,I,L - merged images of TUNEL/GFP and DAPI staining. All images are in dorsal view with anterior towards the right bottom corner.
(8.17 MB TIF)

Figure S3 Cell proliferation in the dorsal hindbrain as detected by anti-pH3 antibody. Two developmental stages were analyzed: 36hpf (A–C Gateways, D–F smu694b/Gateways) and 48hfp (G–I Gateways, J–L smu694b/Gateways). A,D,G,J - anti-pH3 antibody staining; B,E,H,K - anti-GFP antibody staining; C,F,I,L - merged images of anti-pH3/GFP staining and DAPI staining. All images are in dorsal view with anterior towards the right bottom corner.
(9.34 MB TIF)

Movie S1 GFP expression in ChP and r5. Movie is a 3D reconstruction of a series of dorsal view z-stacks taken from a 72hpf Gateways embryo. Red label: Cell TraceTM Bodipy TR methyl ester. Note the difference in morphology between the dorsal ChP cells and the lateral radial cells in r5. Rotation starts with anterior to the left.
(2.70 MB MOV)

Movie S2 In vivo development of ChP in Gateways transgenic zebrafish (I). First phase in ChP development when the tela choroidea appears at the roof of the fourth ventricle. Some cells are located at the dorsal midline of the hindbrain when we first detect them (yellow traced ones) while others appear later in the rhombic lips and delaminate towards the midline (pink traced cells). As the intensity of detection was set to detect early weak expression of GFP at the point of its appearance, by the end of the recording period the intensity of fluorescence went above the upper limit of sensitivity resulting in appearance of a red “over the threshold” staining of ChP cells. The movie represents a single recording period.
(1.28 MB MOV)

Movie S3 In vivo development of ChP in Gateways transgenic zebrafish (II). Second phase in ChP morphogenesis. The major developmental event is the recruitment of new cells into the tela choroidea from the dorsal edges of rhombic lips (pink traced cells) and slightly deeper positions (green arrows). During this phase GFP-positive cells initiate subtle movements and are getting closer to one another. These movements differ from the active movements of another population of GFP-positive cells “patrolling” the border of the roof of fourth ventricle along the rhombic lips (red dot traced cells). The movie represents a single recording period.
(2.76 MB MOV)

Movie S4 In vivo development of ChP in Gateways transgenic zebrafish (III). Third and last phase of ChP morphogenesis. Final transformation of the tela choroidea into the ChP proper by means of the coalescence of cells in the dorsal midline. Note that ChP occupies a territory with clear A-P limits at the level of the ear and separated from the GFP-negative region of the cerebellum (blue trace). The movie is composed of two consecutive recording periods from the same embryo.
(2.01 MB MOV)

Movie S5 In vivo development of ChP in mibtfi91 mutant transgenic zebrafish (I). This movie is composed of two time-lapse recordings made one after the other from the same embryo. It spans the first and second phases of ChP morphogenesis which are greatly impaired. An increased accumulation of GFP expression in the rhombic lips illustrates a reduced ability of ChP cells to detach and move towards the midline. A few of the GFP-positive cells still divide (orange traced cells) unlike what happens in normal development.
(2.32 MB MOV)

Movie S6 In vivo development of ChP in mibtfi91 mutant transgenic zebrafish (II). Third phase of ChP morphogenesis. An impaired coalescence of ChP cells gives rise to a patchy and misplaced ChP. GFP-negative domains interrupt the field of GFP-positive cells in the roof of fourth ventricle that is closely connected to cells of the spinal cord roof plate and cerebellum (blue trace) indicating the inability to maintain its A-P limits at the level of the ear. The movie consists of two time-lapse series recorded from the same embryo one after the other.
(1.83 MB MOV)

Movie S7 In vivo development of ChP in smu649b mutant transgenic zebrafish (I). First and second phases in ChP morphogenesis recorded in a single recording session. Only a narrow stripe of ChP cells is present at the dorsal midline and there is no cell contribution from the rhombic lips. Moreover several cells show fragmentation in manifestation of apoptosis (purple arrows) unlike normal ChP development.
(2.06 MB MOV)

Movie S8 In vivo development of ChP in smu649b mutant transgenic zebrafish (II). Last phase of ChP development. Apoptotic events can still be detected (purple arrow), ChP cells are bigger and their number is reduced compared to normal embryos. Coalescence of cells is less obvious and GFP-positive cells are less compact. This movie is a composite of two time-lapse series recorded one after the other from the same embryo.
(0.86 MB MOV)

## References

[1] Johanson C, Michael Conn P (2003). The Choroid Plexus–CSF Nexus. Gateway to the Brain.. Neuroscience in Medicine.

[2] Redzic ZB, Preston JE, Duncan JA, Chodobski A, Szmydynger-Chodobska J (2005). The choroid plexus-cerebrospinal fluid system: from development to aging.. Curr Top Dev Biol.

[3] Dziegielewska KM, Ek J, Habgood MD, Saunders NR (2001). Development of the choroid plexus.. Microsc Res Tech.

[4] Netsky MG, Shuangshoti S (1970). Studies on the choroid plexus.. Neurosci Res (N Y).

[5] Sturrock RR (1979). A morphological study of the development of the mouse choroid plexus.. J Anat.

[6] Altman J, Bayer SA (1980). Development of the brain stem in the rat. I. Thymidine-radiographic study of the time of origin of neurons of the lower medulla.. J Comp Neurol.

[7] Strong LH (1956). Early development of the ependyma and vascular pattern of the fourth ventricular choroid plexus in the rabbit.. Am J Anat.

[8] Turkevich NG (1963). Embryonic Development of the Vascular Plexus of the 4th Ventricle and of the “Spongy Organ” in Man.. Arkh Anat Gistol Embriol.

[9] Jacobsen M, Clausen PP, Jacobsen GK, Saunders NR, Møllgård K (1982). Intracellular plasma proteins in human fetal choroid plexus during development. I. Developmental stages in relation to the number of epithelial cells which contain albumin in telencephalic, diencephalic and myelencephalic choroid plexus.. Brain Res.

[10] Hunter NL, Dymecki SM (2007). Molecularly and temporally separable lineages form the hindbrain roof plate and contribute differentially to the choroid plexus.. Development.

[11] Landsberg RL, Awatramani RB, Hunter N, Farago AF, DiPietrantonio HJ (2005). Hindbrain rhombic lip is comprised of discrete progenitor cell populations allocated by Pax6.. Neuron.

[12] Chizhikov VV, Millen KJ (2005). Roof plate-dependent patterning of the vertebrate dorsal central nervous system.. Dev Biol.

[13] Lametschwandtner A, Laminger A, Adam H (1983). Development and differentiation of the brain ventricular system in tadpoles of Xenopus laeris (Daudin) (Amphibia, Anura).. Z Mikrosk Anat Forsch.

[14] Cserr HF, Bundgaard M (1984). Blood-brain interfaces in vertebrates: a comparative approach.. Am J Physiol.

[15] Tsuneki K (1986). A survey of occurrence of about seventeen circumventricular organs in brains of various vertebrates with special reference to lower groups.. J Hirnforsch.

[16] Hansson E, Lendahl U, Chapman G (2004). Notch signaling in development and disease.. Sem Cancer Biol.

[17] Chandrasekhar A (2004). Turning heads: development of vertebrate branchiomotor neurons.. Dev Dyn.

[18] Cayuso J, Ulloa F, Cox B, Briscoe J, Marti E (2006). The Sonic hedgehog pathway independently controls the patterning, proliferation and survival of neuroepithelial cells by regulating Gli activity.. *Development*.

[19] Ingham PW, Placzek M (2006). Orchestrating organogenesis: variations on a theme by sonic hedgehog.. Nat Rev Genet.

[20] Awatramani R, Soriano P, Rodríguez C, Mai JJ, Dymecki SM (2003). Cryptic boundaries in roof plate and choroid plexus identified by intersectional gene activation.. Nat Genet.

[21] Dang L, Fan X, Chaudhry A, Wang M, Gaiano N (2006). Notch3 signaling initiates choroid plexus tumor formation.. Oncogene.

[22] Franz T (1994). Extra-toes (Xt) homozygous mutant mice demonstrate a role for the Gli-3 gene in the development of the forebrain.. Acta Anat (Basel).

[23] Higuchi M, Kiyama H, Hayakawa T, Hamada Y, Tsujimoto Y (1995). Differential expression of Notch1 and Notch2 in developing and adult mouse brain.. Brain Res Mol Brain Res.

[24] Irvin DK, Nakano I, Paucar A, Kornblum HI (2004). Patterns of Jagged1, Jagged2, Delta-like 1 and Delta-like 3 expression during late embryonic and postnatal brain development suggest multiple functional roles in progenitors and differentiated cells.. J Neurosci Res.

[25] Varga ZM, Amores A, Lewis KE, Yan Y-L, Postlethwait JH (2001). Zebrafish smoothened functions in ventral neural tube specification and axon tract formation.. Development.

[26] Chen W, Burgess S, Hopkins N (2001). Analysis of the zebrafish smoothened mutant reveals conserved and divergent functions of hedgehog activity.. Development.

[27] Ke Z, Kondrychyn I, Gong Z, Korzh V (2008). Combined activity of the two Gli2 genes of zebrafish play a major role in Hedgehog signaling during zebrafish neurodevelopment.. Mol Cell Neurosci.

[28] Jiang YJ, Brand M, Heisenberg CP, Beuchle D, Furutani-Seiki M (1996). Mutations affecting neurogenesis and brain morphology in the zebrafish, Danio rerio.. Development.

[29] Itoh M, Kim C-H, Palardy G, Oda T, Jiang Y-J (2003). Mind Bomb is a ubiquitin ligase that is essential for efficient activation of notch signaling by Delta.. Dev Cell.

[30] Ke Z, Emelyanov A, Lim S, Korzh V, Gong Z (2005). Expression of a novel zebrafish zinc finger gene, *gli2b*, is affected in Hedgehog and Notch signaling related mutants during embryonic development.. Dev Dynam.

[31] Wang X, Emelyanov A, Korzh V, Gong Z (2003). Zebrafish atonal homologue zath3 is expressed during neurogenesis in embryonic development.. Dev Dyn.

[32] Korzh V (2007). Transposons as tools for enhancer trap screens in vertebrates.. Genome Biol.

[33] Parinov S, Kondrichin I, Korzh V, Emelyanov A (2004). Tol2 transposon-mediated enhancer trap to identify developmentally regulated zebrafish genes in vivo.. Dev Dyn.

[34] Choo BG, Kondrichin I, Parinov S, Emelyanov A, Go W (2006). Zebrafish transgenic Enhancer TRAP line database (ZETRAP).. BMC Dev Biol.

[35] Bill B, Balciunas D, McCarra J, Young E, Xiong T The zebrafish (*Danio rerio*) as a model for vertebrate choroid plexus development.. PLoS One (in press).

[36] Gingras S, Pelletier S, Boyd K, Ihle JN (2007). Characterization of a family of novel cysteine- serine-rich nuclear proteins (CSRNP).. PLoS ONE.

[37] Ishiguro H, Tsunoda T, Tanaka T, Fujii Y, Nakamura Y (2001). Identification of AXUD1, a novel human gene induced by AXIN1 and its reduced expression in human carcinomas of the lung, liver, colon and kidney.. Oncogene.

[38] Joly J-S, Osorio J, Alunni A, Auger H, Kano S (2007). Windows of the brain: towards a developmental biology of circumventricular and other neurohemal organs.. Semin Cell Dev Biol.

[39] Hashimoto PH (1992). Blood-brain barrier and cerebrospinal fluid circulation.. Kaibogaku Zasshi.

[40] Isogai S, Horiguchi M, Weinstein BM (2001). The vascular anatomy of the developing zebrafish: an atlas of embryonic and early larval development.. Dev Biol.

[41] Bingham S, Chaudhari S, Vanderlaan G, Itoh M, Chitnis A (2003). Neurogenic phenotype of mind bomb mutants leads to severe patterning defects in the zebrafish hindbrain.. Dev Dyn.

[42] Baehr C, Reichel V, Fricker G (2006). Choroid plexus epithelial monolayers–a cell culture model from porcine brain.. Cerebrospinal Fluid Res..

[43] Fong S, Emelyanov A, Teh C, Korzh V (2005). Wnt signaling mediated by Tbx2b regulates cell migration during formation of the neural plate.. Development.

[44] Steinberg MS, Takeichi M (1994). Experimental specification of cell sorting, tissue spreading, and specific spatial patterning by quantitative differences in cadherin expression.. Proc Natl Acad Sci USA.

[45] Szmydynger-Chodobska J, Chung I, Chodobski A (2006). Chronic hypernatremia increases the expression of vasopressin and voltage-gated Na channels in the rat choroid plexus.. Neuroendocrinol.

[46] Estrach S, Legg J, Watt FM (2007). Syntenin mediates Delta1-induced cohesiveness of epidermal stem cells in culture.. J Cell Sci.

[47] Jeong J, Mao J, Tenzen T, Kottmann AH, McMahon AP (2004). Hedgehog signaling in the neural crest cells regulates the patterning and growth of facial primordial.. Genes Dev.

[48] Pons S, Trejo JL, Martínez-Morales JR, Martí E (2001). Vitronectin regulates Sonic hedgehog activity during cerebellum development through CREB phosphorylation.. Development.

[49] Wechsler-Reya RJ (2001). Caught in the matrix: how vitronectin controls neuronal differentiation.. Trends Neurosci.

[50] Lundberg F, Li DQ, Falkenback D, Lea T, Siesjö P (1999). Presence of vitronectin and activated complement factor C9 on ventriculoperitoneal shunts and temporary ventricular drainage catheters.. J Neurosurg.

[51] Lam CS, Sleptsova-Friedrich I, Munro AD, Korzh V (2003). SHH and FGF8 play distinct roles during development of noradrenergic neurons in the locus coeruleus of the zebrafish.. Mol Cell Neurosci.

[52] Westerfield M (1993). The Zebrafish Book; A Guide for the Laboratory Use of Zebrafish (Brachydanio rerio)..

[53] Korzh V, Sleptsova-Friedrich I, Liao J, He J, Gong Z (1998). Expression of zebrafish bHLH genes *ngn1* and *nrD* define distinct stages of neural differentiation.. Dev Dynam..

[54] Berghmans S, Murphey RD, Wienholds E, Neuberg D, Kutok JL (2005). *tp53* mutant zebrafish develop malignant peripheral nerve sheath tumors.. Proc Natl Acad Sci U S A.

