# Supplementary figures and images for: In vivo Analysis of Choroid Plexus Morphogenesis in Zebrafish
Source: PLoS One. 2008 Sep 1;3(9):e3090. doi: 10.1371/journal.pone.0003090 (PMC2525818; doi:10.1371/journal.pone.0003090)

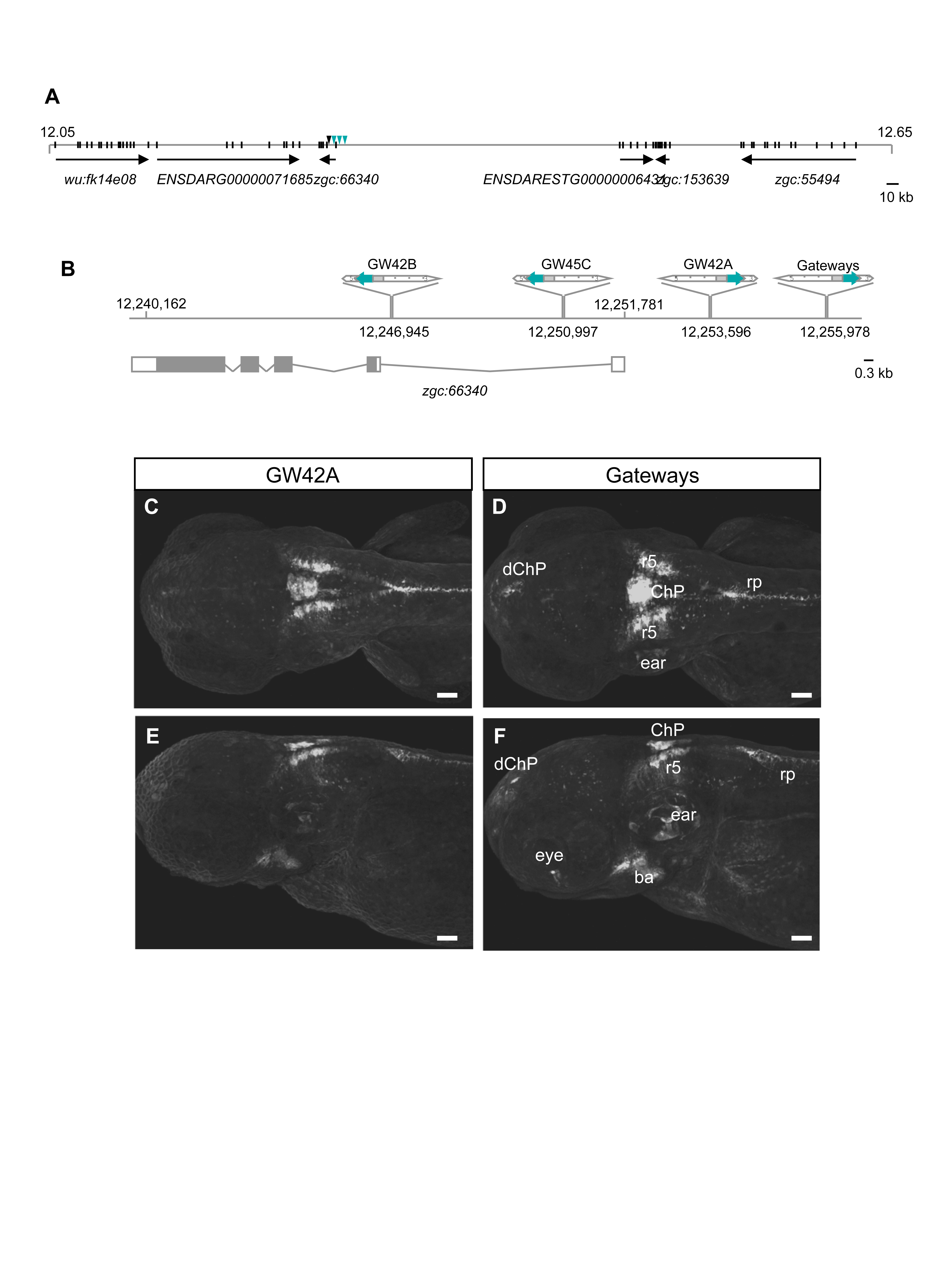

Supplement: Figure S1 — Characterization of the Gateways Tol2 insertion site. (A) 600-kb locus of Chr. 24 (Zv7, release 49). Triangles represent ET insertions, where green triangles show insertions with GFP expression in ChP. (B) zgc:66340 locus. TSS at 12251781 bp was determined using 5′-RACE. The coding exons are depicted as black boxes, untranslated regions are represented as open boxes. The ET insertions are shown according to their position in zgc:66340 locus, green arrow shows EGFP gene in the ET construct (direction of the reporter gene transcription corresponds to the direction of arrow), gray box is a minimal promoter, dotted arrows represent 5′- and 3′-ends of Tol2 transposon). ET, enhancer trap; TSS, transcription start site; EGFP, enhanced GFP. (C,D, C',D') Dorsal and lateral views of GW42A and Gateways lines with insertions located in promoter region at different distances from TSS exhibit GFP expression in ChP. GW45C line with insertion located in intron has a weak GFP expression in ChP (not shown), while another intron-based insertion GW42B shows background GFP expression in skin (not shown). Abbreviations: ba - branchial arch, dChP - diencephalic ChP; ChP - choroid plexus of hindbrain, r5 - rhombomere 5, rp - roof plate. (2.50 MB TIF) [file pone.0003090.s001.tif]

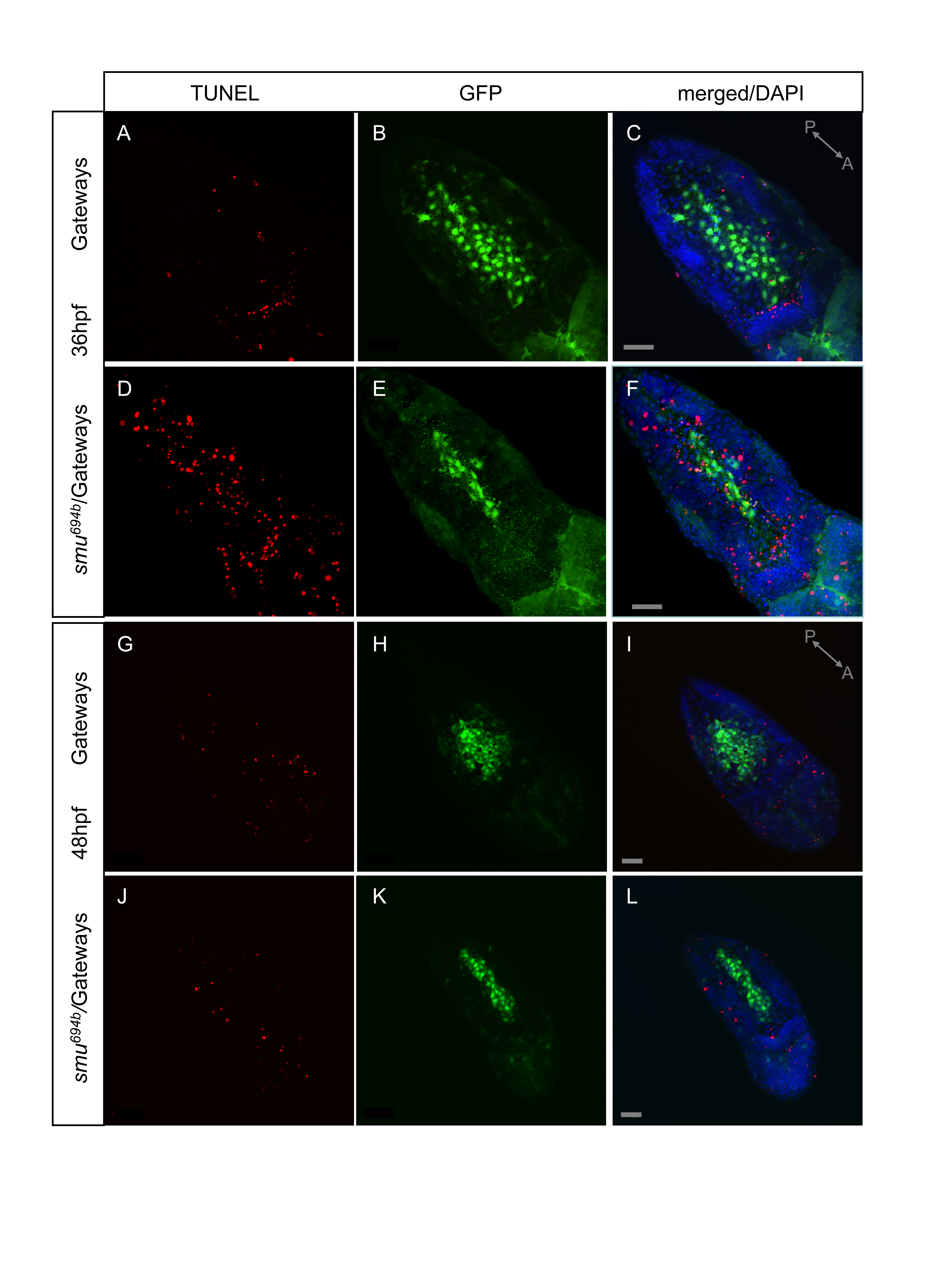

Supplement: Figure S2 — Apoptosis increased in smu mutant as detected by TUNEL. Two developmental stages were analyzed: 36hpf (A–C Gateways, D–F smu694b/Gateways) and 48hfp (G–I Gateways, J–L smu694b/Gateways). A,D,G,J - TUNEL; B,E,H,K - anti-GFP antibody staining; C,F,I,L - merged images of TUNEL/GFP and DAPI staining. All images are in dorsal view with anterior towards the right bottom corner. (8.17 MB TIF) [file pone.0003090.s002.tif]

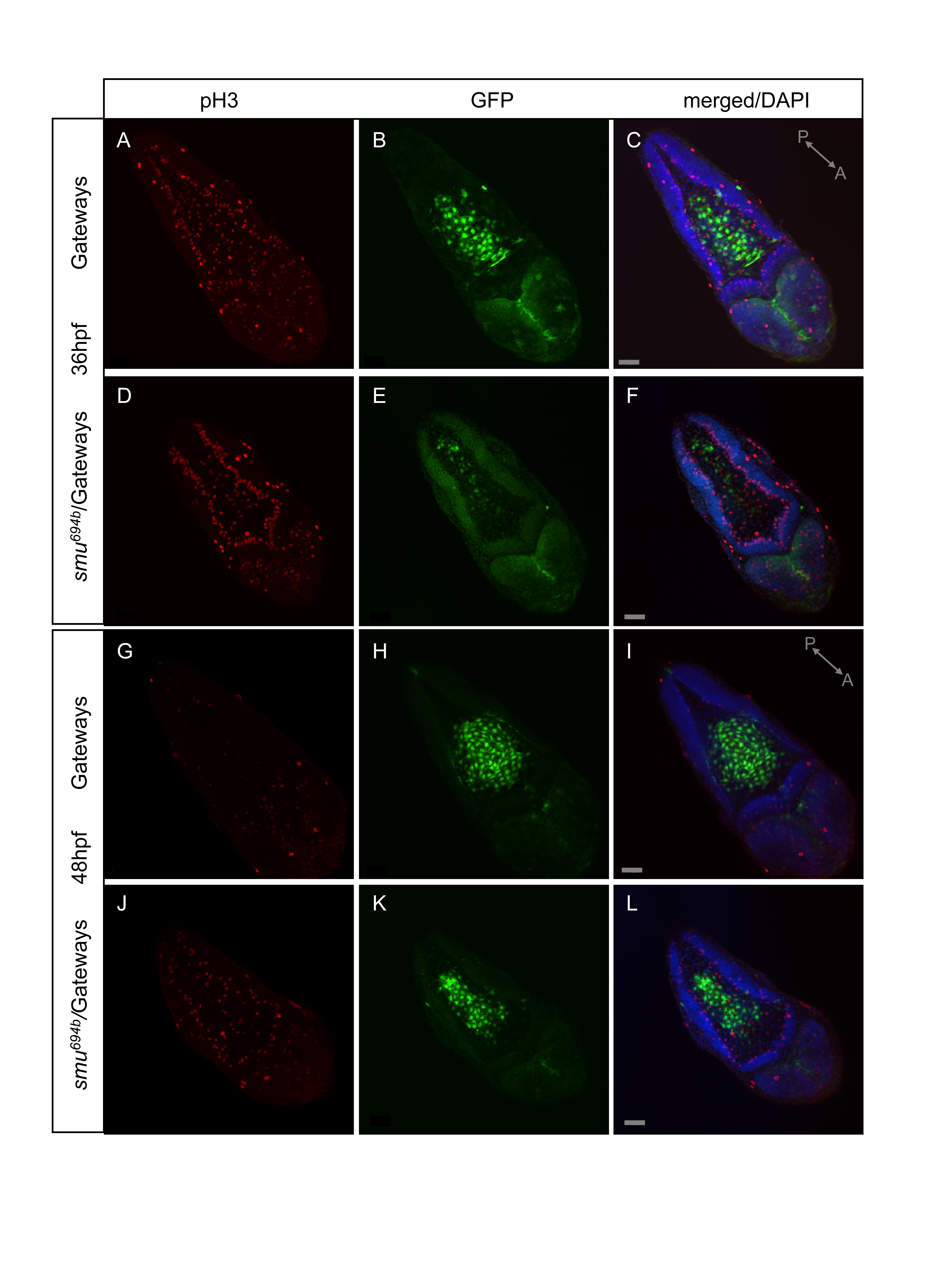

Supplement: Figure S3 — Cell proliferation in the dorsal hindbrain as detected by anti-pH3 antibody. Two developmental stages were analyzed: 36hpf (A–C Gateways, D–F smu694b/Gateways) and 48hfp (G–I Gateways, J–L smu694b/Gateways). A,D,G,J - anti-pH3 antibody staining; B,E,H,K - anti-GFP antibody staining; C,F,I,L - merged images of anti-pH3/GFP staining and DAPI staining. All images are in dorsal view with anterior towards the right bottom corner. (9.34 MB TIF) [file pone.0003090.s003.tif]
